# Computational modelling of acetabular morphology and its implications for cup positioning

**DOI:** 10.3389/fbioe.2025.1629271

**Published:** 2025-07-28

**Authors:** Sara De Angelis, Johann Henckel, Alister Hart, Anna Di Laura

**Affiliations:** ^1^Department of Mechanical Engineering, University College London, London, United Kingdom; ^2^ Royal National Orthopaedic Hospital NHS Trust, Stanmore, United Kingdom; ^3^Institute of Orthopaedics and Musculoskeletal Science, University College London, London, United Kingdom; ^4^ Cleveland Clinic London, London, United Kingdom

**Keywords:** acetabulum, hip joint, statistical shape modelling, anatomical variation, principal component analysis, cup positioning, personalised safe zone

## Abstract

Achieving accurate cup positioning in total hip arthroplasty (THA) remains challenging due to the variable orientation and complex morphology of the bony acetabulum relative to the pelvis. Statistical shape modelling (SSM) has been used to describe the pelvic morphological differences that exist between sexes. However, the effect of these differences on the orientation of the cup/acetabular component in THA has not yet been investigated. The research questions this study aimed to address were i. What are the anatomical variations of the innominate bone between sexes? and ii. Do these sex-based differences have an effect on the position of the acetabular component of a hip replacement? Two sex-specific models were built on three-dimensional (3D) representations of 100 healthy bony hemipelvises (50 female and 50 male hemipelvises) which were generated from pelvic computed tomography (CT) images. Principal component analysis (PCA) was implemented to identify the main components of anatomical variation within each group, the principal components (PCs). Variability in size, shape as well as acetabular orientation of the innominate bone was found in both sex-based models. Four and five PCs accounted for 90% of the cumulative variance for the male and female models, respectively. Acetabular orientation was identified as one of the main PCs, supporting the indication that the variability commonly found in the orientation of a prosthetic acetabular component (inclination and version) is influenced by the anatomical shape of the native acetabulum. A better understanding of the relationship between innominate bone morphology and cup positioning can help plan the orientation of acetabular prosthetic components more accurately and define more personalised safe zones. Patient-specific models based on acetabular geometry can enable individualised surgical planning, potentially reducing the risk of postoperative complications such as dislocation, wear and joint instability.

## 1 Introduction

Achieving the surgical target for acetabular cup orientation represents a challenge in total hip arthroplasty (THA). Optimal positioning of this component is essential to ensure joint stability, optimise range of motion, and avoid complications. Malpositioning of the cup can cause impingement (Yamaguchi et al., 2000), dislocation (Biedermann et al., 2005) and wear of the bearing surfaces (Patil et al., 2003), affecting the hip biomechanics (Kiyama et al., 2009).

Several studies (Callanan et al., 2010; Grammatopoulos et al., 2015; Bosker et al., 2007; Hassan et al., 1998) have reported that the percentage of the components being positioned outside of what is considered the ‘safe zone’ (40° ± 10° for inclination and 15° ± 10° for anteversion) (Lewinnek et al., 1978) is high, ranging from 20% to 70%. Amongst the causes is the variable relative orientation of both the pelvis and acetabulum (Beverland et al., 2016). Therefore, the ‘safe zone’ only represents a guide for cup positioning (Abdel et al., 2015; Reina et al., 2017; Danoff et al., 2016). A better understanding of the innominate bone shape could help define a more effective safe zone for acetabular cup orientation in THA.

Statistical shape modelling (SSM) has been employed to analyse pelvic anatomical variations and investigate sex-related differences (Arand et al., 2018; Ahrend et al., 2020; van Veldhuizen et al., 2023). Arand et al. (2018) and Ahrend et al., (2020) identified statistically significant differences in the distance between the anterior inferior iliac spines (AIIS) and the conjugate vera, respectively. van Veldhuizen et al. (2023) reported that the most pronounced differences were found in the iliac wing and pubic rami regions. However, the impact of these pelvic morphological variations on acetabular cup positioning in total hip arthroplasty remains unexplored.

The aim of this study was to better understand the shape variations of the innominate bone that may influence the positioning of acetabular prosthetic components. Our primary objective was to use SSM to identify the main modes of morphological variation in the innominate bone with a focus on differences between sexes.

## 2 Materials and methods

### 2.1 Data preparation

A total of 67 pre-operative pelvic computed tomography (CT) scans of Caucasian patients (42 males and 25 females) were analysed. The mean age was 66 years old (range: 33 to 92, standard deviation (SD): 13). All 25 female patients had non-diseased pelvises. Amongst the 42 male patients, 8 had non-diseased pelvises while 34 had unilateral osteoarthritis (OA); in those cases, only the non-diseased side was included in the analysis. Institutional review board approval NHS RNOH R&D Service Evaluation (SE16.020–11/08/2016).

Two sex-specific hemipelvis models were built, each comprising 50 hemipelvises. Digital Imaging and Communications in Medicine (DICOM) CT files were imported into Simpleware ScanIP Medical (Version 2024.6; Synopsys Inc. Mountain View, CA), where three-dimensional (3D) models of each pelvis were generated using an AI-based segmentation tool. To validate segmentation accuracy, a subset of 20 pelvises was manually segmented using thresholding techniques and compared to the AI-generated segmentations. This comparison yielded an average Dice similarity coefficient of 0.95, indicating high segmentation accuracy. For the threshold-based segmentations, soft and hard tissues were separated by setting the Hounsfield (HU) window for bone between 230:3020 HU (Singh et al., 2022). The pixels of interest formed a mask corresponding to the regions of interest. The mask was then separated into multiple ones in order to isolate the hemipelvises and exclude the femurs and the sacrum. Any gaps and holes present on the final masks were then filled to refine the segmentations. On the other hand, the AI-based segmentation tool automatically detected the anatomical regions of interest (ROIs) and separated them accordingly. This way the hemipelvises could be isolated without the need for manual cropping, improving reproducibility and requiring no manual cleanup. As all CT scans had a slice thickness ≤1 mm, image resolution was deemed sufficient to minimise segmentation artefacts.

The anterior pelvic plane (APP) (Lewinnek et al., 1978) defined by the right and left anterior iliac spines (ASISs) and the pubic tubercle (PT) was used as the anatomical reference plane. Python scripting was implemented to realign the global coordinate system to the anatomical one standardising all models to the APP.

**FIGURE 1:**
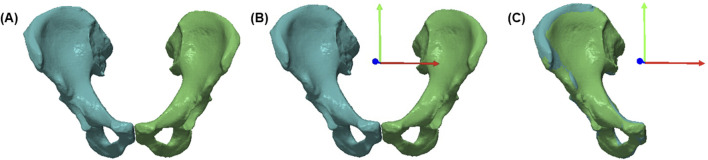
**(A)** Segmentation of pelvis, **(B)** Realignment of global axis to anatomical axis (APP), **(C)** Mirroring of left hemipelvis with respect to the APP.

To generate the two sex-specific innominate bony models, all left hemipelvises were mirrored, resulting in two training sets of 50 right hemipelvises each. Mirroring was achieved by modifying the transformation matrix of the surface mesh to invert the scaling factor along the global x-axis, effectively flipping the geometry relative to the standardised reference frame, Figure 1. For each sex-specific model, the following analysis steps were subsequently performed.

### 2.2 Statistical shape model

#### 2.2.1 Initial alignment and mean shape generation

All hemipelvises were imported into a common file for processing. Firstly, a Gaussian recursive filter (sigma = 2) was applied to each surface to reduce mesh roughness and smooth out surface irregularities. Secondly, they were manually aligned to ensure a consistent spatial orientation across all samples. Each surface in the dataset was defined by a vector *X*_*i*_ consisting of a number of points *p*_*j*_ defined by their x, y, and z coordinates across the surfaces (Lorenz and Krahnstöver, 2000), Equation 1.

$$X_{i}=\left[\left(p_{jx},p_{jy}{,p}_{jz}\right)\right]$$ (1)

with *i* = 1 … 50 and *j* = 1 … *N* (*i*) where *N* is the number of points of surface *i*th.

 Following alignment, point correspondence was established to create isotopological meshes with uniform node distribution. A mean shape $\bar{X}$ was calculated by averaging the discrete points across all *n* training surfaces in the dataset, Equation 2.

$$\bar{X}=\frac{1}{n}\sum_{i=1}^{n}X_{i}$$ (2)

**FIGURE 2:**
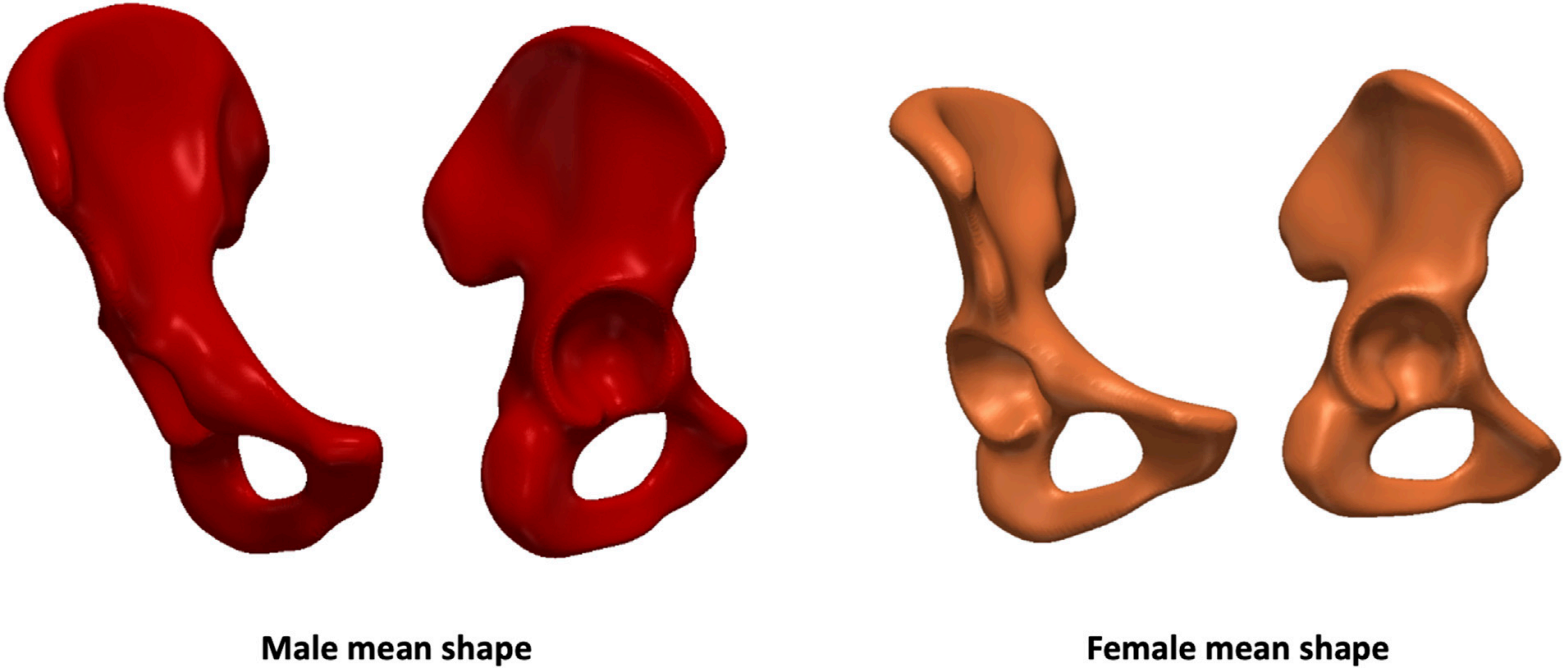
Male and female mean shapes generated from each sex-specific model.

The mean shapes generated from each sex-specific model are shown in Figure 2 below.

#### 2.2.2 Point mapping

**FIGURE 3:**
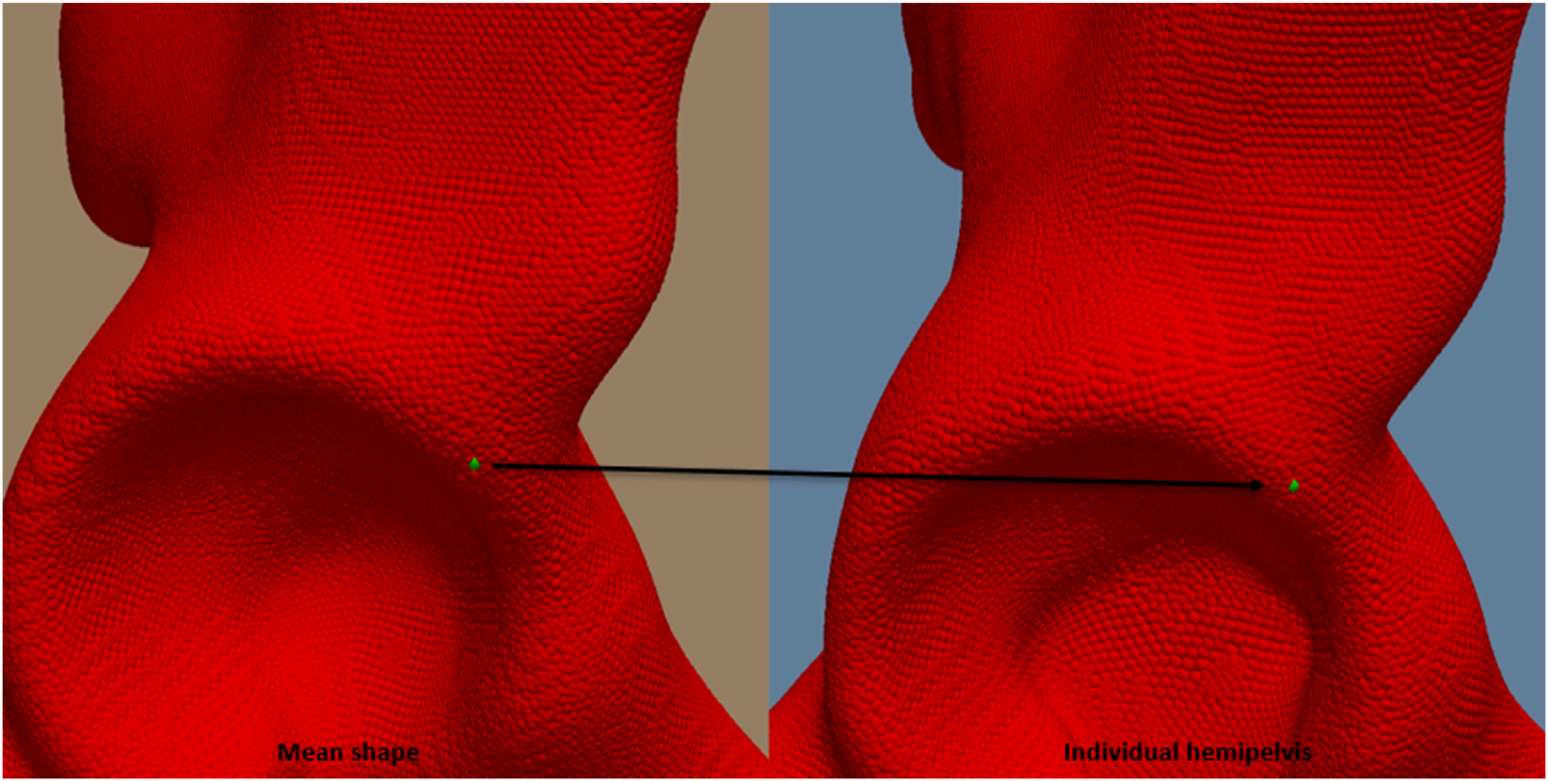
Point mapping between each hemipelvis and the mean shape of one of the discrete landmarks.

The target mean shape was mapped to each individual hemipelvis and the vertex positions of the target defined the correspondences across all shapes, Figure 3. This allowed the model to capture the relative positions of these points and identify patterns of anatomical shape variability among patients.

#### 2.2.3 Principal component analysis

Principal component analysis (PCA) was implemented to identify the main shape variations within the dataset. This dimensionality reduction technique identifies the directions of maximum variance known as principal components (PCs) or modes of variation. Given a dataset representing a certain shape deformation, PCA is performed by decomposing the covariance matrix of the sample data using a matrix factorisation technique called singular value decomposition (Wang et al., 2017). The principal components correspond to the eigenvectors $\vec{x\;}$ of the covariance matrix, while the eigenvalues *λ* represent the amount of variance carried by each PC. The PCs are ranked according to their contribution to the total variance, with the first few components typically capturing the most significant shape variations. The sample covariance matrix C, Equation 3, is defined as:

$$C=\frac{1}{n-1}\sum_{i=1}^{n}\left(x_{i}-\bar{x}\right){\left(x_{i}-\bar{x}\right)}^{T}$$ (3)

where *x*_*i*_ represents each sample in the dataset and $\bar{x}$ is the sample mean vector.

### 2.3 Model evaluation

Following the application of PCA, each principal component (PC) was visualized as ± SDs. Plots of individual and cumulative explained variance (model compactness) were generated to determine how many components were necessary to capture the majority of shape variation in the dataset. Only PCs accounting for more than 5% of the total variance were included in the analysis. The principal components were visually inspected by a team of engineers and orthopaedic surgeons to identify key anatomical features for further investigation. Feature-based measurements were then performed to objectively assess the relationship between each principal component (PC) and the corresponding anatomical variations. Shape variations of the innominate bone were assessed by calculating changes in the anatomical parameters identified during visual inspection across all modes of variation. The mean shape was systematically deformed by adjusting the weight of each PC from −3 SD to +3 SD. Measurements were taken at the extreme points: -3 SD and +3 SD. This allowed us to evaluate how the surfaces deviated from the mean. The range (difference) between the measurements was calculated to quantify the spread in the data that corresponded to each mode. For each mode, the anatomical feature exhibiting the greatest range was identified as the most prominently affected. In cases where multiple features exhibited similar levels of variation (i.e., within one degree of difference), all were considered equally prominent for that mode. Finally, the mean shapes of both models (e.g., male and female, if implied) were compared to highlight key morphological differences in relation to the identified anatomical parameters.

### 2.4 Statistical analysis

Sex-based differences in anatomical parameters were assessed using independent Student’s t-tests, with a significance threshold set at p < 0.05. To test the intra-observer and inter-observer reproducibility of the feature-based measurements, the intra-class correlation coefficient (ICC) was calculated. A two-way mixed absolute agreement model was used and average measures ICC values were reported along with 95% confidence intervals (CI).

## 3 Results

### 3.1 Model evaluation

#### 3.1.1 Male model evaluation

**FIGURE 4:**
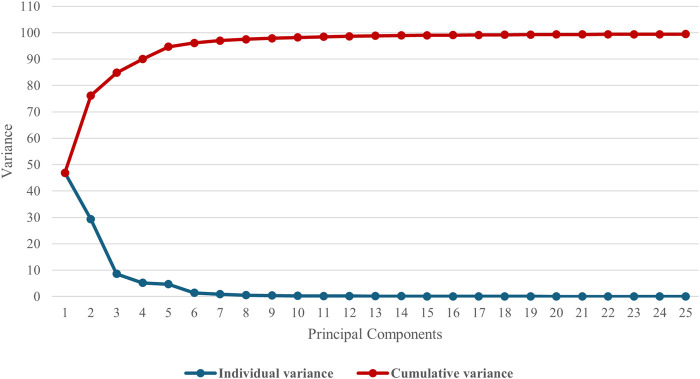
Individual and cumulative variance plot showing 90% of the cumulative variance was captured by 4 principal components for the **male** model.

Four modes of shape variation accounted for 90% of the variance described by the male model as shown in Figure 4. This was considered sufficient to encompass the most relevant features of the innominate bone, since the individual variance explained by mode 5 and subsequent components was below 5%, leading to their exclusion from further analysis. The modes are presented in order of their contribution to the overall dataset variance and are discussed below.

**FIGURE 5:**
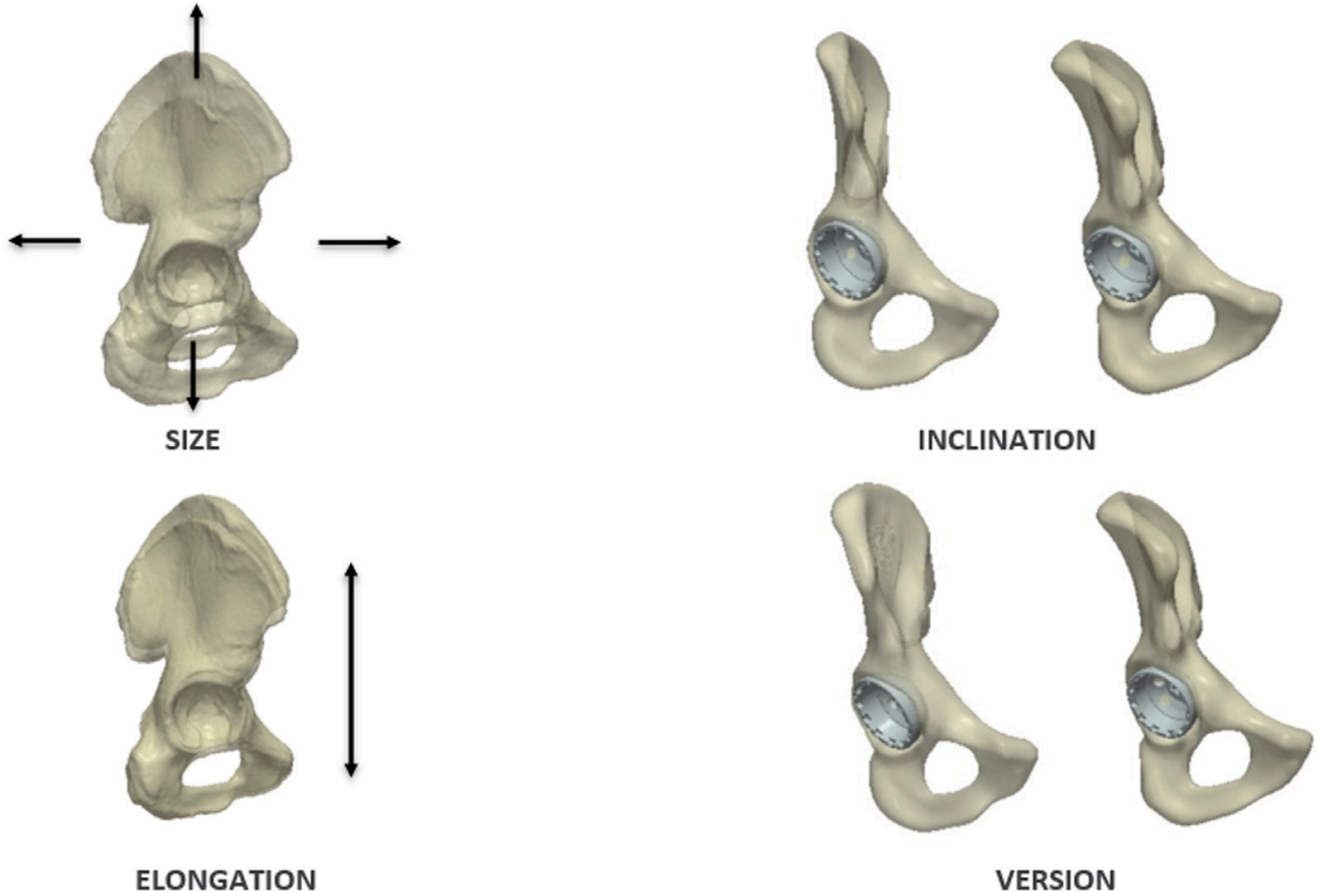
Anatomical features analysed in the study: size, elongation, inclination and version.

**FIGURE 6:**
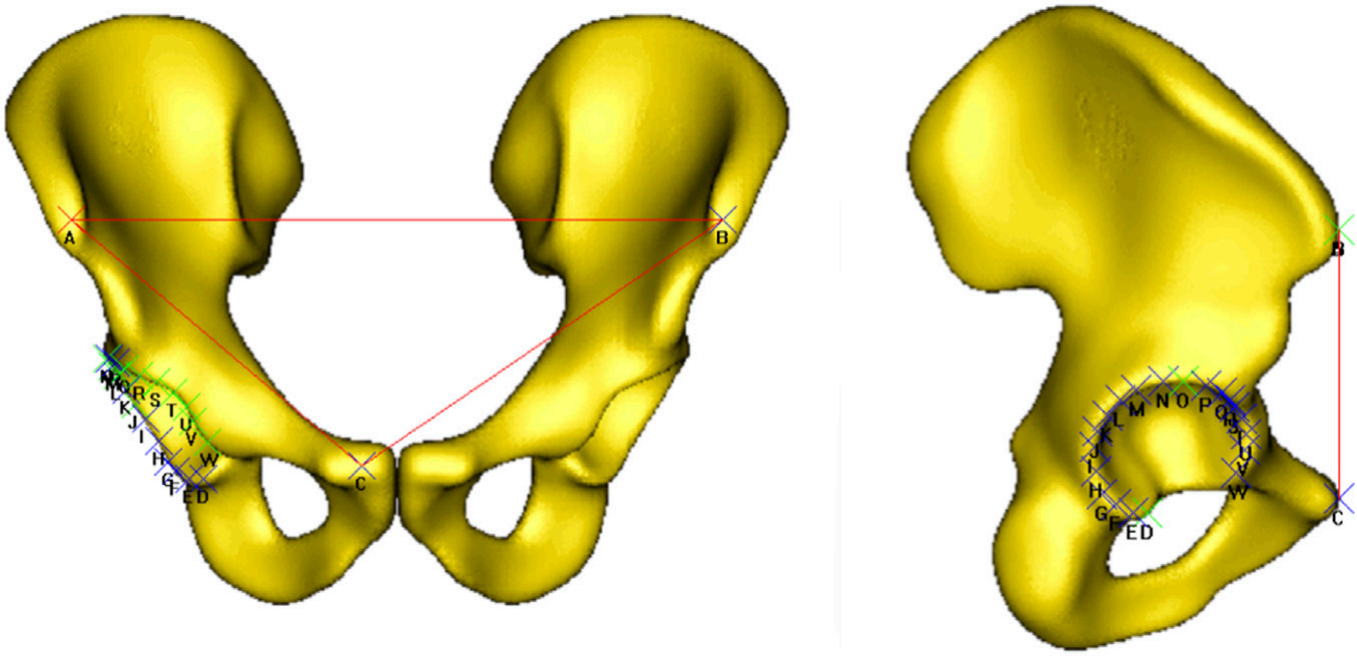
Method used to calculate inclination and version angles. 20 points were placed around the rim of the acetabulum excluding the notch, with respect to the APP.

The anatomical features analysed included: size, elongation, inclination and version; Figure 5. These features were chosen to correlate the SSM results to known clinical parameters. Specifically, acetabular rotation was defined in terms of version and inclination relative to the pelvic coordinate system. While radiographic inclination is defined as the angle between the longitudinal axis of the body and the acetabular axis when projected onto the coronal plane, radiographic version is the angle between the acetabular axis and the coronal plane (Murray, 1993). Having realigned all training surfaces to the APP during the data preparation process, pelvic tilt became constant across all samples and was no longer a source of variation. Size was assessed through volumetric measurements. Elongation was quantified by measuring the distance between the hip joint centre and the superior notch of the iliac crest. To evaluate acetabular orientation, each shape was mirrored to standardise the anterior pelvic plane (APP). A standard landmarking protocol was employed placing 20 points along the acetabular rim excluding the notch (Murtha et al., 2008; Vandenbussche et al., 2008; Osmani et al., 2013; Higgins et al., 2014; Zhang et al., 2017; Hua and Li, 2022). Inclination and version angles (radiographic definition (Murray, 1993)) were then computed relative to the APP using Robin’s 3D imaging software (Robin’s 3D 3.3.8.0); Figure 6.

**FIGURE 7:**
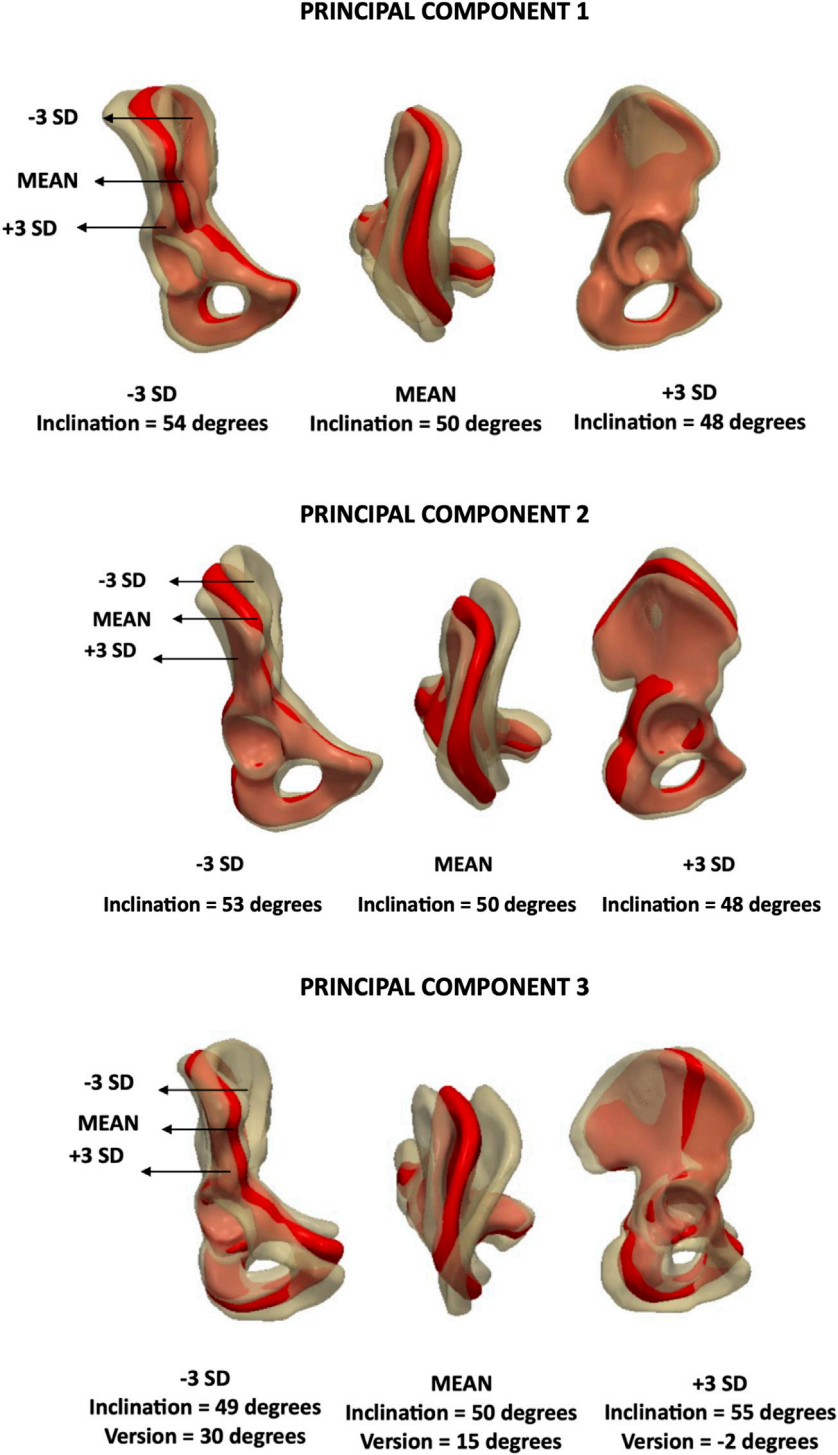
Analysis of PC1/PC2/PC3 – **male** model. Anteroposterior, axial and lateral views of the mean shape in red, along with ±3 SDs surfaces showing changes in inclination and version.

The first principal component, PC1, accounted for the greatest variance. PC1 described variations in acetabular inclination and accounted for 47% of anatomical variation (eigenvalue = 1.1 × 10^7^). PC2 also captured changes in inclination and accounted for 29% of the variance (eigenvalue = 6.9 × 10^6^). The third principal component, PC3, was associated with changes in acetabular version and inclination. The individual variance of this mode was 9% (eigenvalue = 2.0 × 10^6^). These results are illustrated in Figure 7.

**FIGURE 8:**
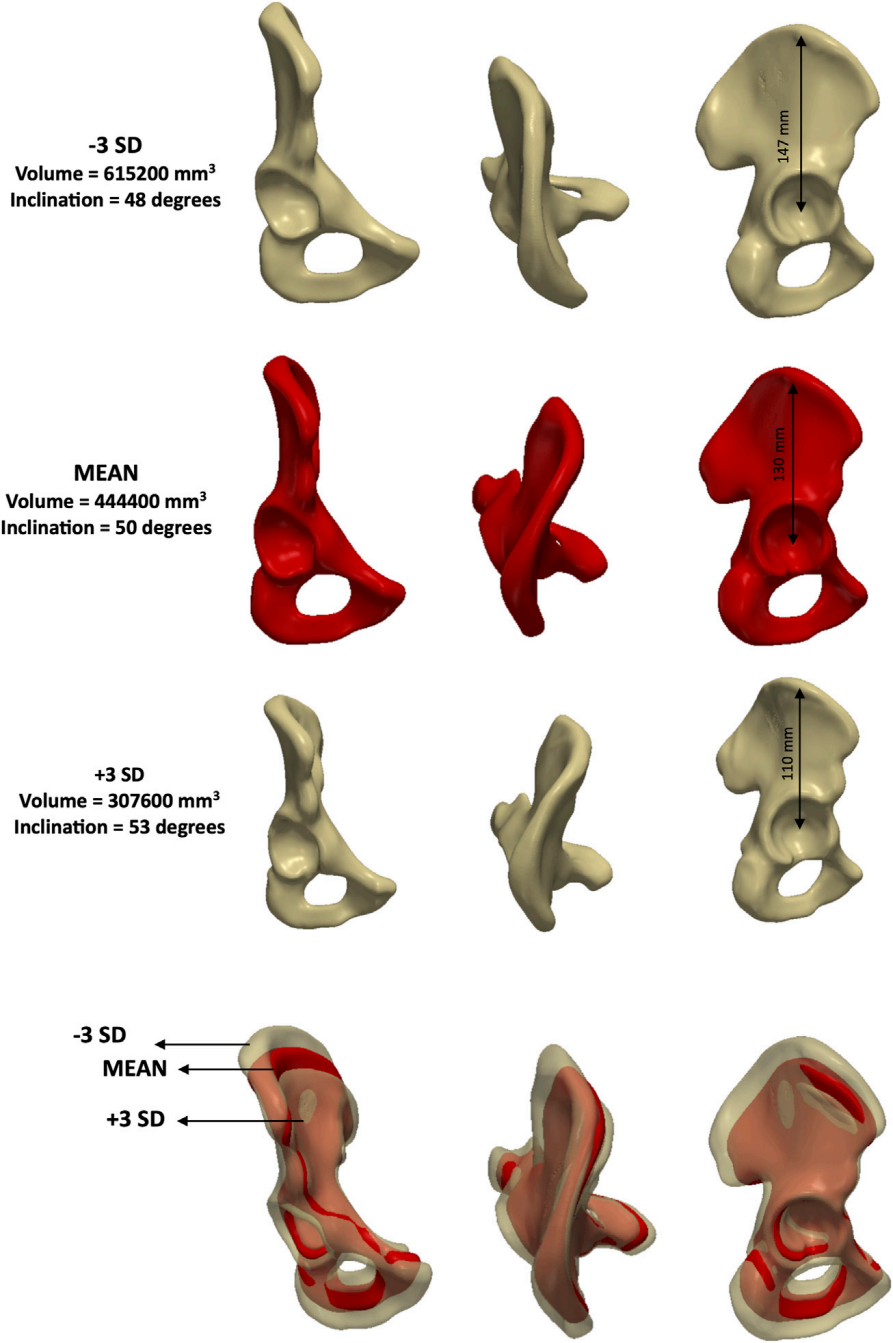
Analysis of PC4 – **male** model. Anteroposterior, axial and lateral views of the mean shape in red, along with ±3 SDs surfaces (top and third row), showing changes in size, elongation and inclination.

Lastly, PC4 represented a combination of size, elongation and inclination (Figure 8) according to the feature-based measurements (volume, distance between hip joint centre and top notch as well as inclination angle). This mode explained 5% of the variance individually (eigenvalue = 1.2 × 10^6^).

#### 3.1.2 Female model evaluation

**FIGURE 9:**
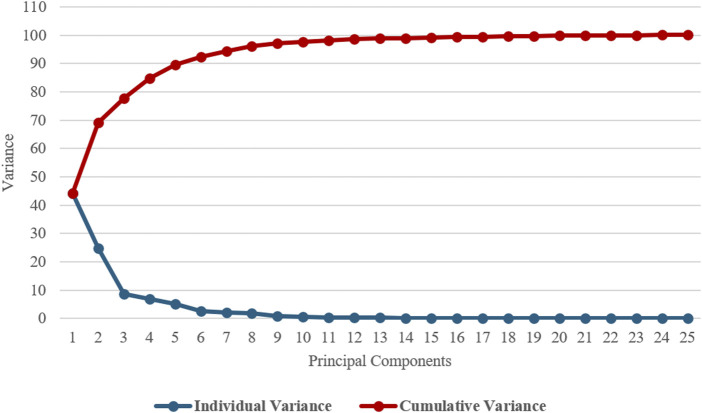
Individual and cumulative variance plot showing 90% of the cumulative variance was captured by 5 principal components for the **female** model.

Five modes accounted for 90% of the total variance in the female model, as illustrated in Figure 9. As for the male model, each anatomical feature - size, elongation, inclination and version - was evaluated across all modes and the PCs that best described each feature were identified.

**FIGURE 10:**
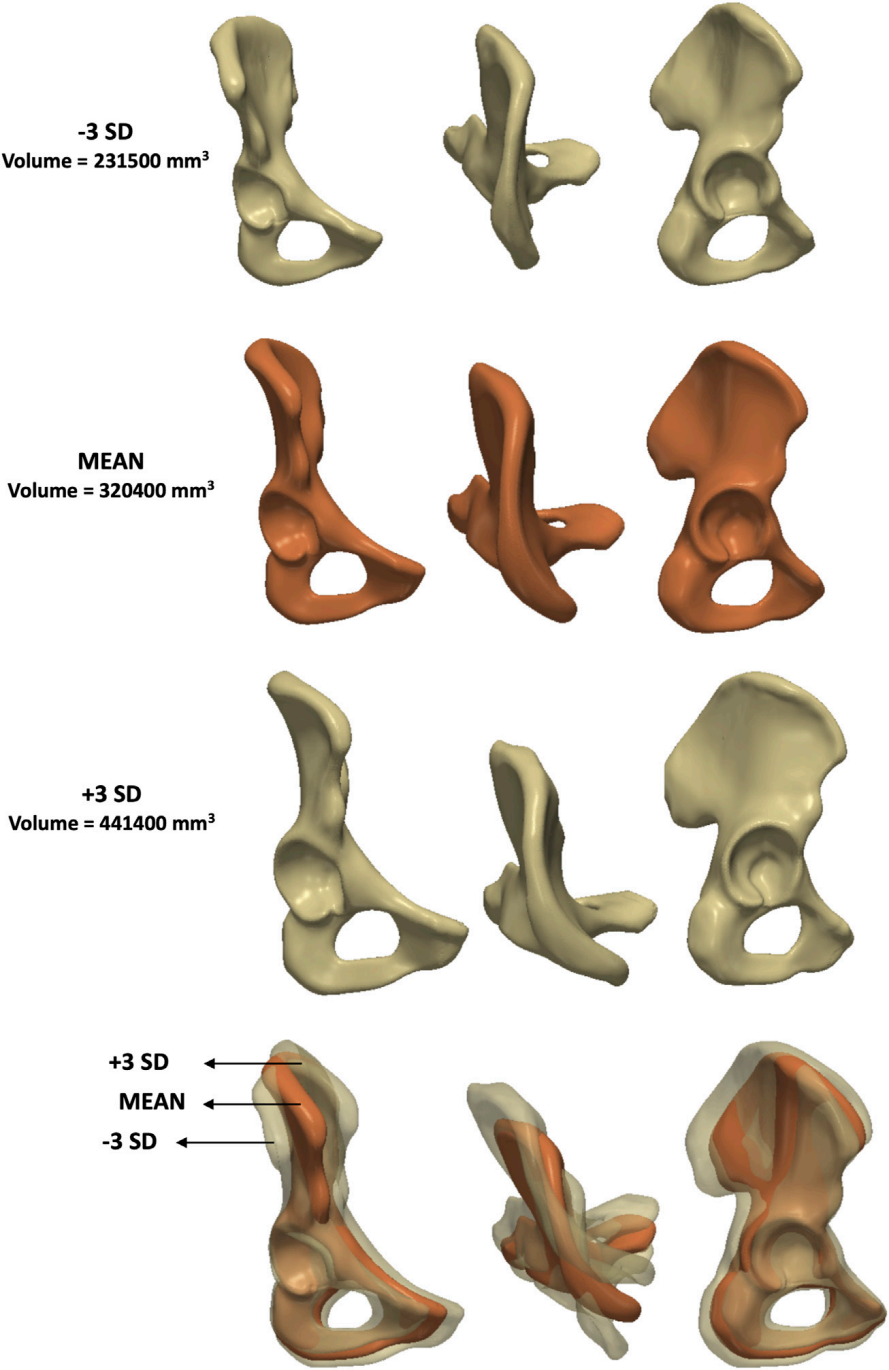
Analysis of PC1 – **female** model. Anteroposterior, axial and lateral views of the mean shape in orange, along with ±3 SDs surfaces (top and third row), showing changes in size.

**FIGURE 11:**
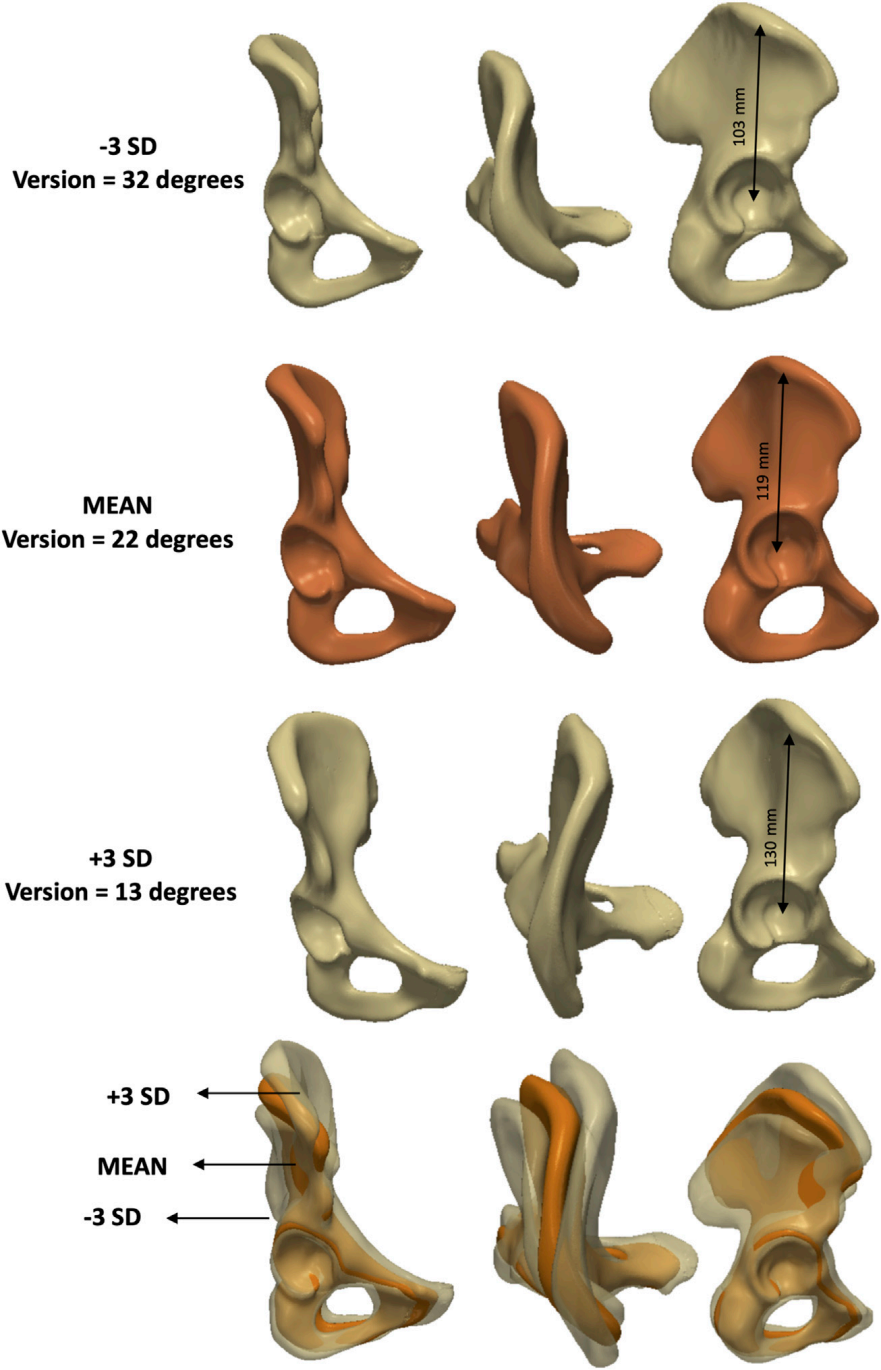
Analysis of PC2 – **female** model. Anteroposterior, axial and lateral views of the mean shape in orange, along with ±3 SDs surfaces (top and third row), showing changes in elongation and version.

The first principal component (PC1) primarily captured variations in size (Figure 10), contributing towards 44% of the total variance (eigenvalue = 4.1 × 10^6^). PC2 accounted for 25% of the variance (eigenvalue = 2.3 × 10^6^) and was associated with changes in both elongation and version (Figure 11).

**FIGURE 12:**
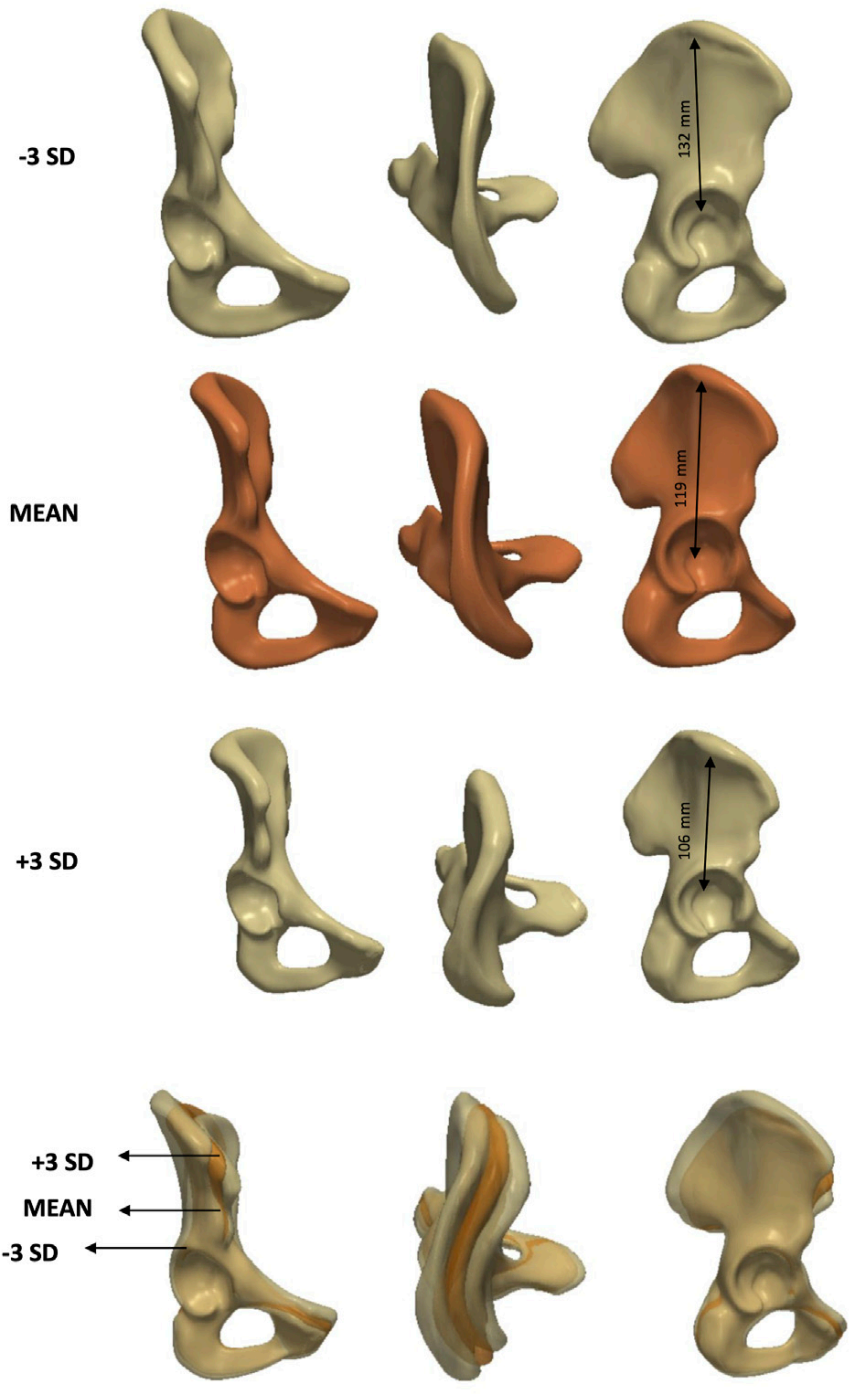
Analysis of PC3 - **female** model. Anteroposterior, axial and lateral views of the mean shape in orange, along with ± 3 SDs surfaces (top and third row) showing changes in elongation.

**FIGURE 13:**
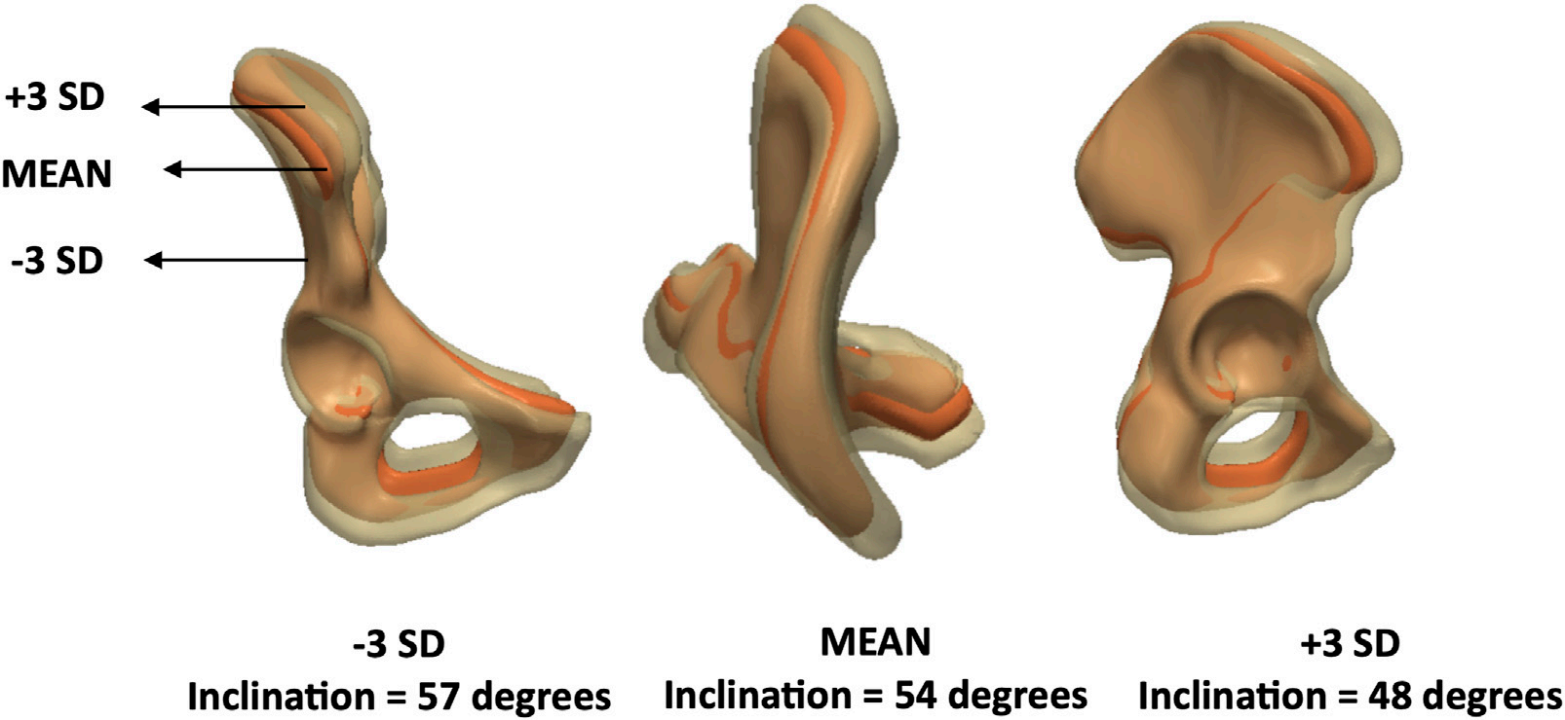
Analysis of PC5 – **female** model. Anteroposterior, axial and lateral views of the mean shape in orange, along with ±3 SDs surfaces showing changes in inclination.

PC3 (Figure 12), which explained 9% of the variance (eigenvalue = 7.9 × 10^5^), was linked to elongation. PC4 represented a more complex mode involving multiple parameters without a single dominant anatomical feature and explained 7% of the variance (eigenvalue = 6.1 × 10^5^). Finally, PC5 was primarily associated with changes in inclination (Figure 13) and accounted for 5% of the variance (eigenvalue = 4.2 × 10^5^).

#### 3.1.3 Mean shapes comparison

The volume of the male mean shape was measured at 444,400 (range: 307,600–615,200) mm^3^, whereas the female mean shape had a volume of 320,400 (range: 231,500–441,400) mm^3^. Mean elongation in males was 130 mm (range: 110–147) compared to 119 mm (range: 103–130) in females. When investigating inclination, the male and female mean shapes reported angles of 50 (range: 48–54) and 54 (range: 48–57) degrees, respectively. Lastly, version angles were 15 degrees (range: −2–30) for males and 22 (range: 13–32) degrees for females.

### 3.2 Statistical analysis

When assessing sex-based differences, pelvic size was found to be statistically significant (p-value = 0.0012). Similarly, elongation was also statistically significant with a p-value of 0.0120. Lastly, while differences in inclination values were non-significant (p-value = 0.1527), a statistically significant difference was found for version (p-value = 0.0360).

Intra- and inter-observer results were calculated for elongation, inclination and version. Size was excluded from the analysis as its computation was automatic and did not involve any subjectivity. When testing for intra-observer reliability, the ICCs for elongation were 0.98 (95% CI 0.85–1.00) for males and 0.98 (95% CI 0.92–1.00) for females. The corresponding coefficients for inclination were 0.99 (95% CI 0.94–1.00) for males and 0.98 (95% CI 0.92–0.99) for females. Lastly, the ICCs for version were 1.00 (95% CI 1.00 to 1.00) and 0.99 (95% CI 0.98–1.00) for males and females, respectively. The inter-observer results showed coefficients of 0.99 (95% CI 0.94–1.00), 0.97 (95% CI 0.86–0.99) and 1.00 (95% CI 0.95–1.00) for male elongation, inclination and version, respectively. In the female cohort, the corresponding coefficients were 0.96 (95% CI 0.85–0.99), 0.94 (95% CI 0.78–98) and 0.99 (95% CI 0.93–1.00), respectively.

## 4 Discussion

The aim of this study was to identify sex-based differences in innominate bone morphology that may influence the positioning of acetabular prosthetic components. Principal component 1 (PC1) primarily captured variations in inclination in males, while in females it reflected differences in overall pelvic size. PC2 was associated with changes in inclination in males and with elongation and version in females. In the male model, PC3 corresponded to variations in acetabular orientation, including both inclination and version, whereas in the female model it was primarily linked to elongation. PC4 reflected variations in size, elongation and inclination in males while it had no dominant anatomical feature in females but rather represented a complex combination of all parameters. Lastly, PC5 exhibited changes in inclination in the female cohort.

It is known that the size of the male pelvis is greater than the female one (Dzupa et al., 2021). The male acetabulum is also larger as it is designed to fit a bigger femur (Leong, 2006). This aligns with the findings of the study as the male mean shape resulted to be much greater than the female in terms of volume. As also stated in the literature, the male pelvis is taller and narrower compared to the female pelvis (Fischer and Mitteroecker, 2017). The female human anatomy is designed for childbearing; therefore, the hip bone is broader and shallower. The pelvic inlet also presents with a larger opening. For these reasons, females have been reported to have lower neck shaft angles (Sariali et al., 2009). This is also shown in the comparison between our male and female mean shapes which showed an elongation of 130 and 119 mm, respectively. A study by Atkinson et al. (2010) analysed sex-based differences in hip morphology on 100 consecutive CT scans of Caucasian patients undergoing hip resurfacing for early osteoarthritis (OA). Acetabular version was found to significantly vary more in female patients, ranging from 10 to 53 degrees, compared to male ones (range: 7–46 degrees). This is also reflected by our model as PC2 (which carried 25% of the variance) and PC3 (9%) accounted for version in females and males, respectively. As version is associated with acetabular coverage, investigating differences between subjects can also be relevant when treating patients with developmental dysplasia of the hip (DDH). DDH is characterised by a deficiency in the anterolateral acetabular coverage of the femoral head. This leads to increased joint reactive forces, an overload of the acetabular rim, and subsequent degeneration of the articular cartilage (Ganz and Leunig, 2007). To correct for acetabular dysplasia deformities, periacetabular osteotomy (PAO) is carried out. During this procedure, the affected bone is surgically cut (osteotomised). The acetabulum is then reoriented to reduce superomedial inclination, improve femoral head coverage, medialise the hip joint centre and normalise the acetabular rim loading (Clohisy et al., 2009; Ganz et al., 1988). Various factors affect the results of PAO, including secondary femoroacetabular impingement (FAI). Duncan et al. (2015) aimed to determine whether sex-specific differences between male and female patients undergoing PAO for acetabular dysplasia affected the outcomes. A greater presence of clinical, radiographic, and intraarticular findings linked to secondary FAI was found in male patients with acetabular dysplasia. Tannenbaum et al. (2014) found the male acetabulum to be significantly more retroverted than the female acetabulum. The pathophysiological basis of acetabular retroversion is an anterior acetabular hyper-coverage of >30°–40° (Direito-Santos et al., 2018). Acetabular retroversion is associated with FAI, which is more common in men than women (O’Rourke and El Bitar, 2023). This is in line with the findings of our study, where the female acetabulum was found to be more anteverted with a mean of 22 (range: 13–32) degrees compared to 15 (range: -2–30) degrees in males. Direct comparison between inclination and version angles calculated in our study and the literature can only be performed with those studies that reported radiographic acetabular orientation measurements (Osmani et al., 2013; Higgins et al., 2014; Zhang et al., 2017; Hua and Li, 2022). Based on our findings, inclination angles were found to be non-significant (p-value = 0.1527) between sexes while version angles were statistically different (p-value = 0.0360). This is in agreement with previous studies (Higgins et al., 2014; Zhang et al., 2017; Hua and Li, 2022).

Although existing studies (Arand et al., 2018; Ahrend et al., 2020; van Veldhuizen et al., 2023) have implemented the use of computational models to investigate pelvic sex-specific differences, they have not investigated the effect of the pelvic morphological variations on the cup orientation. They have also not standardised for pelvic tilt. Setting the reference frame to the APP makes the results more clinically relevant for preoperative planning purposes.

This study offers a better understanding of the innominate bone shape in relation to cup position. The nature of optimal cup positioning is multifactorial. Every patient’s pelvis has unique anatomical differences, such as variations in acetabular inclination and version. The planning and execution of acetabular reorientation procedures rely on an understanding of each patient’s unique anatomical characteristics (Govsa et al., 2005). SSM may offer the surgeon a guide to appreciate these variations preoperatively and aid cup position, which plays a crucial role in minimising impingement and dislocation risks. (Scheerlinck, 2014). This may help to plan the orientation of acetabular cups in THA more accurately, improving hip joint biomechanics and force distribution within the joint.

This study presents limitations. Firstly, the models were built on 100 Caucasian hemipelvises. In order to capture more morphological variations of the innominate bone, a higher number of subjects should be included. Different ethnic groups should also be investigated to provide a more accurate representation of the global population. We acknowledge that the training dataset used in the study represents a specific population, and as such, the resulting reference model may not be fully generalisable to all possible populations or clinical subgroups. Additionally, while the dataset accounted for key demographic variables (Merrill et al., 2018), other covariates such as physical activity level, comorbidities, or socioeconomic factors were not uniformly available and thus could not be evaluated. Secondly, PCs that accounted for less than 5% were not included in the study. However, they could be helpful in identifying any additional distinguishing features in the innominate bone and could be investigated as part of future work. Thirdly, only 8 non-diseased male pelvises (16 hemipelvises) were used. The remaining 34 hemipelvises were segmented from the non-diseased side of patients with unilateral OA. However, literature shows that in unilateral OA, the unaffected side can maintain normal morphology and biomechanics (Ritter et al., 1996). For comparative analysis, an average shape was computed from the bilateral non-diseased hemipelvises and compared to that derived from the non-diseased hemipelvises of patients with unilateral OA. A Dice similarity coefficient of 0.91 was found suggesting high but not perfect shape correspondence. While the results still need to be interpreted with caution, this finding indicates a measurable shape bias introduced by using contralateral hips of OA patients. Additionally, isolating shape geometry independent of scale could further enrich the findings by removing this variable as a source of variation. However, as the primary objective of the present study was to investigate sex differences accounting for both shape and size variations, with a view towards virtual anatomical reconstruction, the surfaces were not scaled in this instance. Lastly, the inclusion of younger patients could improve our understanding of developmental hip morphology and support more tailored treatment strategies across different age groups. As part of future work, the SSM-derived anatomical shape variation could be combined with multibody models to simulate personalised movement and/or loading as well as understand the correlation between morphology and function (Putame et al., 2019; Zhang et al., 2025).

## 5 Conclusion

This study evaluated inter-subject anatomical variability of the innominate bone and its effect on cup component positioning. Variations in size, shape and orientation were captured by a series of PCs. The model showed significant variability in acetabular orientation across individuals providing a better understanding of sex-based pelvic morpho-structural differences. These findings enhance our understanding of sex-based morpho-structural differences in the pelvis and highlight the need for individualized considerations in cup positioning. Such insights may inform surgical planning, particularly in the context of patient-specific instrumentation and robotic systems, where accurate component alignment is critical.

## Data Availability

The datasets presented in this article are not readily available because of patient confidentiality. Requests to access the datasets should be directed to anna.laura.14@ucl.ac.uk.
